# Impact of a Mediterranean diet on hepatic and metabolic outcomes in non‐alcoholic fatty liver disease: The MEDINA randomised controlled trial

**DOI:** 10.1111/liv.15264

**Published:** 2022-04-26

**Authors:** Elena S. George, Anjana Reddy, Amanda J. Nicoll, Marno C. Ryan, Catherine Itsiopoulos, Gavin Abbott, Nathan A. Johnson, Siddharth Sood, Stuart K. Roberts, Audrey C. Tierney

**Affiliations:** ^1^ Institute for Physical Activity and Nutrition, School of Exercise and Nutrition Sciences Deakin University Geelong Australia; ^2^ School of Allied Health, Human Services and Sport La Trobe University Australia; ^3^ Department of Gastroenterology Eastern Health Box Hill Australia; ^4^ Department of Gastroenterology and Hepatology St Vincent’s Hospital Fitzroy Australia; ^5^ School of Health and Biomedical Sciences RMIT University Melbourne Australia; ^6^ The Boden Collaboration for Obesity, Nutrition, Exercise and Eating Disorders The University of Sydney Sydney New South Wales Australia; ^7^ Department of Gastroenterology Melbourne Health Melbourne Australia; ^8^ Department of Gastroenterology Alfred Health Prahran Australia; ^9^ Central Clinical School Monash University Clayton Australia; ^10^ School of Allied Health, Health Implementation Science and Technology Centre, Health Research Institute University of Limerick Ireland

**Keywords:** dietary patterns, intrahepatic lipids, low‐fat diet, Mediterranean diet, non‐alcoholic fatty liver disease, non‐alcoholic steatohepatitis

## Abstract

**Background:**

Non‐alcoholic fatty liver disease (NAFLD) is predominantly managed by lifestyle intervention, in the absence of effective pharmacotherapies. Mediterranean diet (MedDiet) is the recommended diet, albeit with limited evidence.

**Aims:**

To compare an ad libitum MedDiet to low‐fat diet (LFD) in patients with NAFLD for reducing intrahepatic lipids (IHL) by proton magnetic resonance spectroscopy (^1^H‐MRS). Secondary outcomes include insulin resistance by homeostatic model of assessment (HOMA‐IR), visceral fat by bioelectrical impedance analysis (BIA), liver stiffness measurement (LSM) and other metabolic outcomes.

**Methods:**

In this parallel multicentre RCT, subjects were randomised (1:1) to MedDiet or LFD for 12 weeks.

**Results:**

Forty‐two participants (25 females [60%], mean age 52.3 ± 12.6 years) were included, 23 randomised to LFD and 19 to MedDiet.; 39 completed the study. Following 12 weeks, there were no between‐group differences. IHL improved significantly within the LFD group (−17% [log scale]; *p* = .02) but not within the MedDiet group (−8%, *p* = .069). HOMA‐IR reduced in the LFD group (6.5 ± 5.6 to 5.5 ± 5.5, *p* < .01) but not in the MedDiet group (4.4 ± 3.2 to 3.9 ± 2.3, *p* = .07). No differences were found for LSM (MedDiet 7.8 ± 4.0 to 7.6 ± 5.2, *p* = .429; LFD 11.8 ± 14.3 to 10.8 ± 10.2 *p* = .99). Visceral fat reduced significantly in both groups; LFD (−76% [log scale], *p* = <.0005), MedDiet (−61%, *p* = <.0005).

**Conclusions:**

There were no between‐group differences for hepatic and metabolic outcomes when comparing MedDiet to LFD. LFD improved IHL and insulin resistance. Significant improvements in visceral fat were seen within both groups. This study highlights provision of dietary interventions in free‐living adults with NAFLD is challenging.

**Abbreviations**
ALP: alkaline phosphatase
ALT: alanine aminotransferase
AST: aspartate aminotransferase
BMI: body mass index
CI: confidence interval
GGT: gamma‐glutamyltransferase
HbA1c: glycohemoglobin
IHL: intrahepatic lipid
LFD: Low‐fat diet
MedDiet: Mediterranean diet
NAFLD: non‐alcoholic fatty liver disease

Lay Summary

This clinical trial contributes to the limited lifestyle interventions in Western countries demonstrating that dietary interventions in free‐living adults with NAFLD are challenging. Improving diet quality appears to be important, as does weight loss in these patients.

## 1 INTRODUCTION

Non‐alcoholic fatty liver disease (NAFLD) is the most common cause of liver disease worldwide, affecting approximately 20%–30% of the adult population.^1^ NAFLD results from an accumulation of fat in the liver which exceeds 5% of total liver weight and occurs in the absence of excessive alcohol consumption.^2^ NAFLD is often referred to as the hepatic manifestation of metabolic syndrome as it tends to occur with one or more risk factors that define the syndrome including insulin resistance, hypertension, obesity and/or hyperlipidaemia.^3^, ^4^ A variety of therapeutic interventions have been proposed for the management of NAFLD; these have predominantly focused on weight reduction using low‐calorie diets, exercise, pharmacotherapy or bariatric surgery, as well as lipid‐lowering drugs and antioxidant supplementation.^5^ In the absence of effective and safe pharmacotherapy, diet and lifestyle interventions remain the first‐line treatment in NAFLD. The most effective treatment to date is weight loss and there seems to be a direct relationship between percentage weight loss and improvement in risk factors.^6^, ^7^, ^8^ In real‐world settings, weight loss continues to be difficult to achieve and even harder to maintain, with long term follow‐up studies confirming that weight loss is generally not sustained.^9^, ^10^

A large body of literature surrounds the benefits of a Mediterranean diet (MedDiet) in conditions such as metabolic syndrome, T2DM and cardiovascular disease; such conditions often co‐exist and have a pathophysiological link with NAFLD.^11^, ^12^ A small but growing body of evidence from randomised controlled trials (RCTs) continues to demonstrate that increased adherence to a MedDiet, in patients with NAFLD can improve intrahepatic lipid (IHL) levels, fibrosis, insulin resistance and other metabolic risk markers.^13^, ^14^, ^15^, ^16^, ^17^ These trials are heterogeneous with varying clinical outcomes, including relatively few with hepatic specific measures. In addition, prescribed dietary interventions within the studies are variable and thus there is lack of consistent high‐quality evidence to support a superior dietary pattern for this patient group.

Mediterranean diet interventions are also largely limited to Mediterranean regions and thus the feasibility and efficacy of achieving dietary adherence in patients with NAFLD from Western, multiethnic populations have not been determined.^18^, ^19^, ^20^ One tightly controlled trial conducted in Australia, which included the full provision of meals, demonstrated adherence to MedDiet‐elicited reversal of NAFLD through reducing IHL (−39%) and improving insulin resistance (−1.7 mmol/L, using HOMA‐IR) in 6 weeks.^14^ Another RCT in free‐living adults with NAFLD in Australia found MedDiet and a low‐fat diet (LFD) both effectively improved hepatic steatosis indicating that improved diet quality regardless of dietary prescription was beneficial.^15^ Still, current dietary guidelines for patients with NAFLD encompass the principles of an LFD, which underpin the National Dietary Guidelines.^21^, ^22^ There is limited evidence supporting the efficacy of LFD for the improvement of hepatic and cardio‐metabolic risk factors in NAFLD. Benefits that have been reported following the provision of LFD are elicited because of the effects of weight loss and not necessarily the effects of the diet itself.^23^, ^24^

Therefore, the aims of this RCT were to compare an ad libitum MedDiet to a LFD in patients with NAFLD by comparing the efficacy of the two dietary patterns in relation to several metabolic parameters including primary outcome IHL, and secondary outcomes, including insulin resistance as determined by homeostatic model of assessment of insulin resistance (HOMA‐IR). In addition, we assessed whether there are health benefits attributed to adhering to these dietary guidelines and prescriptions in the absence of weight loss.

## 2 MATERIALS AND METHODS

### 2.1 Study design and participant recruitment

**FIGURE 1:**
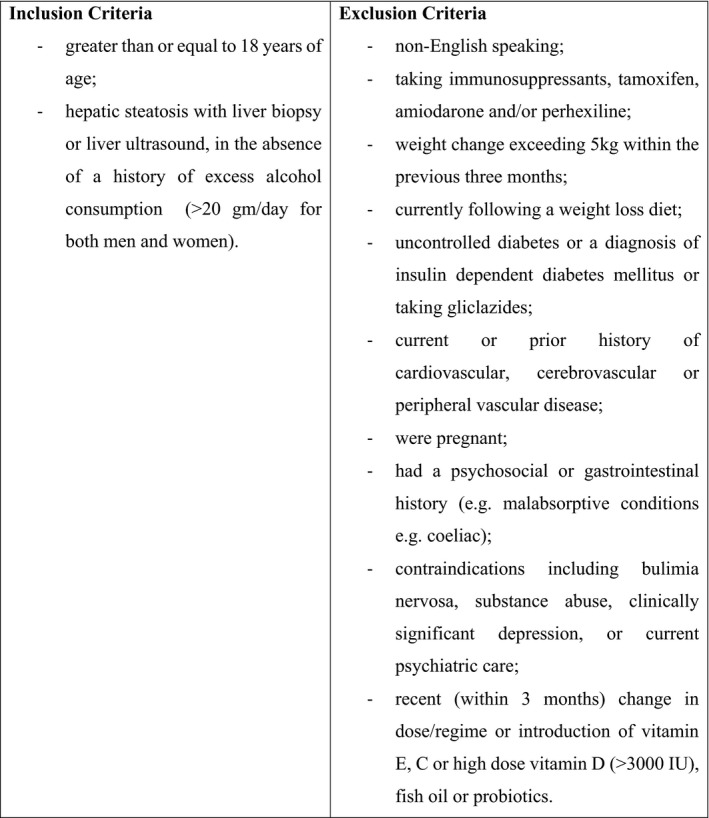
MEDINA study inclusion and exclusion criteria

This study is a parallel multicentre randomised controlled trial registered with the Australian New Zealand Clinical Trials Registry (ANZCTR) Trial ID: ACTRN12615001010583 and was conducted at three large academic liver centres in Melbourne, Australia with recruitment from January 2015 until March 2018, the final appointment was completed in April 2019, 12‐month sustainability data was collected but is not reported herein. Recruitment ceased when the number of participants needed for the intrahepatic lipid’s primary outcome was reached. The published protocol details the study design and methods.^25^ Participants deemed eligible for the study following a screening and baseline visit conducted by a researcher were enrolled in the study. Participant eligibility included those aged >18 years who had a body mass index (BMI) between 20 and 39.9 kg/m^2^, with a diagnosis of NAFLD (without any evidence of other forms of liver disease), determined by routine ultrasound or biopsy; insulin resistance based on a HOMA IR score of >2; and at least one elevated serum ALT level (>20 U/L female, >30 U/L male) during the past 6 months. Exclusion criteria are detailed elsewhere.^25^ Following informed consent and screening, participants were randomised 1:1, stratified by gender and presence of diabetes by computer‐generated methodology. The random allocation sequence was completed by the statistician who assigned participants to one of the two groups. Participants had to meet the inclusion criteria defined in Figure 1. Eligible participants identified from liver clinic appointment lists who were interested in participating were provided with participant information and consent forms and written consent was obtained. As a result of the nature of the study which prescribed a diet to both groups the participants and dietitians administering the diets could not be blinded. However, all analysis was conducted by a statistician who was blinded to the study conditions. Human research ethics committee approval was obtained from all hospital sites. Approval was also obtained from La Trobe University human research ethics committee.

### 2.2 Diet intervention

The LFD was based on dietary recommendations as set out by the Australian Dietary Guidelines and The Heart Foundation, advising dietary patterns that equated to approximately 30% of total energy from fat, 50% from carbohydrate and 20% from protein.^21^, ^22^ The MedDiet arm was based on a traditional Cretan diet composed of approximately 44% of energy from fat (>50% monounsaturated fatty acids), 33% from carbohydrates, 15%–20% from protein and up to 5% from alcohol, as described by George et al. where qualitative food group targets and example resources have also been described in detail.^26^ Advice regarding alcohol consumption was to remain below 20 g per day as recommended by the National Health and Medical Research Council (NHMRC).^27^ Participants in both dietary intervention groups had three face to face appointments, at baseline and weeks 6 and 12, and an additional three phone call follow up reviews at weeks 2, 4 and 9. Participants randomised to the LFD arm received $20 AUD Coles supermarket vouchers at each face‐to‐face appointment to be spent on key staple foods outlined in the dietary recommendations. Participants randomised to ad libitum MedDiet were given hampers including extra virgin olive oil and nuts for the intervention duration, as well as canned fish and legumes, to model the diet and showcase examples of appropriate staple foods. MedDiet participants were also supplied a Mediterranean diet cookbook.^28^ Dietary data were collected using 3‐day food diaries to determine habitual diet and to assess adherence to dietary prescription. These were collected at baseline, 6 weeks and 12 weeks. Food diaries were self‐reported written records of food and beverage intake. Independent dietitians delivered the respective interventions. Three‐day food diaries were entered and analysed using the software FoodWorks 8™. Dietary interventions overlapped with some of the recommendations provided such as increasing wholegrains, vegetables, fruits and reducing discretionary items.

### 2.3 Demographic and anthropometric data

Demographic information including age, gender, smoking status, education, employment and ethnicity was collected by self‐report at baseline. Anthropometric measures including height (m), weight (kg), waist, hip and neck circumference (cm) were taken at baseline, mid 6 weeks and 12 weeks. Body composition measurements were taken using Bioelectrical Impendence Analysis (BIA) with the Seca mBCA 515. As participants arrived in a fasted state for pathology these always occurred at about the same time, in the morning, participants were asked to void their bladder and wear light clothing, and the same clothing, at all appointments. Any jumpers or jackets were removed as were shoes and socks for the analysis. These data points were collected by a researcher at all face‐to‐face time points; baseline, 6 weeks and 12 weeks. All data collection was in accordance with the research protocol outlined elsewhere.^25^ Height and weight were used to calculate BMI (kg/m^2^).

### 2.4 Primary outcome

Intrahepatic lipids (IHL) were assessed at baseline and at 12 weeks. IHL was measured using non‐invasive proton magnetic resonance spectroscopy (^1^HMRS) on an Avanto 1.5 T system (Siemens) by an independent qualified radiographer, using a protocol developed for the MEDINA trial and detailed previously.^14^ Briefly, hepatic spectra were acquired using point resolved spectroscopy (TR = 3000 ms, TE = 35 ms, 16 measurements, 1024 sample points) with a 3.0 × 3.0 × 3.0 cm volume of interest, during free breathing. IHL was quantified as the percentage of the methylene resonance to water (jMRUI version 5.2, EU Project) by a blinded researcher. This replaced insulin resistance as the primary outcome (as per trial registration) as a result of slow participant recruitment.

### 2.5 Secondary outcomes

Liver stiffness measurement (LSM), in kPa using Fibroscan™ (Echosens) was determined at baseline and 12 weeks by experienced hepatologists at The Alfred who were blinded to the study intervention. All other secondary outcomes were collected at baseline, 6 weeks and 12 weeks and included Homeostatic Model Assessment‐Insulin Resistance (HOMA‐IR),^29^ liver function tests (alanine aminotransferase (ALT), aspartate aminotransferase (AST), gamma‐glutamyl transferase (GGT) and alkaline phosphatase (ALP)), systolic and diastolic blood pressure (mmHg), and serum lipids (total cholesterol (TC), low‐density lipoprotein (LDL), high‐density lipoprotein (HDL) and triglycerides (TGs) (mmol/L)). The inflammatory marker high sensitivity C‐reactive protein (hs‐CRP) (mg/L) was also measured. Reference ranges for these biomarkers were derived from Alfred Health pathology.

### 2.6 MEDAS score

The MEDAS score is a 14 point checklist developed and validated by researchers for the PREDIMED trial, a study which assessed MedDiet in participants with cardiovascular disease.^30^ This score is designed to assess MedDiet adherence where participants received a score of one for adherence and zero for non‐adherence for each of the 14 criteria where a higher score is indicative of greater adherence.^31^ A nine‐point score for adherence to LFD was also developed for the PREDIMED study and used to assess compliance in this study. These scores were used to determine participant adherence to the respective diets and were assessed at baseline and 12 weeks.

### 2.7 Statistical analysis

Descriptive statistics are presented as mean ± standard deviation for continuous data, and frequency and percentage for categorical data. Between‐group intervention effects were estimated using restricted maximum‐likelihood linear mixed models using outcome data from all available time points. These models included terms to estimate group effects at the mid‐ (where available) and post‐intervention time points and were adjusted for pre‐intervention diabetes status and baseline visceral fat level by including their interactions with time point. Specification of an unstructured residual error variance–covariance matrix ensured that models implicitly adjusted for the baseline level of the outcome.^32^ All participants with post‐baseline outcome data contributed to the estimation of intervention effects. A sensitivity analysis was also conducted in which observations that had disproportionate effects on model estimates (as determined by inspection of DFBETA values) were checked for and, where present, the model was refitted with the influential observation(s) omitted. Within‐group changes from baseline to post intervention were estimated separately for each intervention arm using restricted maximum‐likelihood linear mixed models with random intercepts for participants and a fixed effect of time. Inspection of preliminary model diagnostics (e.g., normality of residuals, homogeneity of variance) led to all but six outcomes (fat mass, systolic blood pressure, diastolic blood pressure, total protein, albumin and globulin) being log‐transformed for use in inferential analyses. For models with log‐transformed outcomes, unstandardised coefficients and their confidence intervals can be exponentiated and subsequently interpreted as estimated multiplicated effects on geometric means.^33^ All statistical analyses were conducted using Stata/SE 16 (StataCorp, TX) and statistical significance was set at *p* < .05. For biomarkers including liver enzymes and lipids, there were gender‐specific reference ranges, however because of the small numbers included in this trial these values were combined for statistical analysis.

### 2.8 Power calculation

The sample size calculation was based on IHL summary data in Table 2 of Ryan et al.,^14^ with the assumption of a 25% change of IHL in the MedDiet group and 5% in the LFD group, resulting in difference of 20% in change of IHL. The inputs required power of at least 80% with type I error = 5%. The required sample size for each group was 17. Allowing for a potential 20% dropout, the required sample size was 21 participants per group.

## 3 RESULTS

**FIGURE 2:**
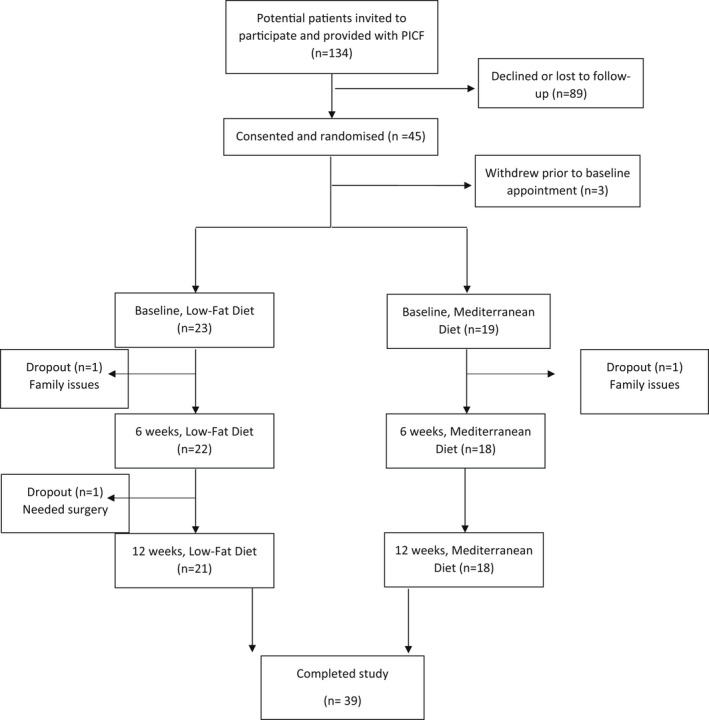
MEDINA participant flow chart

Forty‐two participants were included and 39 completed the study. A flow diagram of participation is shown in Figure 2. Of these 42 participants, 60% were female and the mean age was 52.3 ± 12.6 years. Eighteen participants had diabetes *n* = 7 in the MedDiet group and *n* = 11 in the LFD group. The cohort was multiethnic with a mean BMI of 32.2 ± 6.2 kg/m^2^ and 43% of participants had type 2 diabetes mellitus. Participants were recruited and randomised to the MedDiet (*n* = 19) or LFD (*n* = 23). The baseline demographic, clinical and biochemistry characteristics are shown in Table 1. At baseline visceral fat, heart rate and glucose were substantially higher in the LFD group compared to the MedDiet group.

**TABLE 1:** Baseline demographics and patient characteristics (by diet group)

	Low‐fat diet (*n* = 23)	Mediterranean diet (*n* = 19)	*p*‐value
Demographics			
Age (years)	52.1 ± 13.6	52.6 ± 11.7	.90
Female *n* (%)	14 (61%)	11 (58%)	.99
Diabetes *n* (%)	11 (48%)	7 (37%)	.86
Ethnicity			
Australian *n* (%)	4 (17.4)	4 (21.1)	.17
European *n* (%)	7 (30.5)	6 (32.0)	.68
Chinese Asian *n* (%)	12 (52.2)	8 (42.1)	.20
Middle Eastern *n* (%)	0	1 (5.3)	—
Anthropometry			
Weight (kg)	89.8 ± 24.4	87.7 ± 21.1	.77
BMI (kg/m^2^)	32.7 ± 6.9	31.6 ± 5.4	.57
WC (cm)	108.7 ± 18.9	105.1 ± 14.7	.64
Body composition			
Fat mass (%)	40.9 ± 7.9	39.1 ± 7.9	.49
Visceral fat (L)	4.4 ± 2.1	3.2 ± 1.5	**.017**
Liver outcomes			
IHL (%)	9.2 ± 10.7	13.7 ± 7.8	**.049**
LSM (kPa)	11.8 ± 14.3	7.8 ± 4.0	.68
Haemodynamic measures			
Systolic BP (mmHg)	127.4 ± 19.2	125.4 ± 12.2	.70
Diastolic BP (mmHg)	83.4 ± 9.8	82.8 ± 7.1	.85
Heart rate (bpm)	77.5 ± 14.4	69.5 ± 9.4	**.042**
Insulin resistance and biomarkers			
HOMA‐IR	6.5 ± 5.6	4.4 ± 3.2	.13
Glucose (mmol/L)	6.7 ± 2.0	5.8 ± 1.6	**.028**
Insulin (mIU/L)	20.0 ± 12.4	16.4 ± 8.9	.34
ALT (U/L)	61.5 ± 37.0	54.1 ± 25.2	.75
AST (U/L)	41.8 ± 21.6	31.8 ± 12.6	.17
GGT (U/L)	126.7 ± 128.8	90.1 ± 74.6	.42
ALP (U/L)	93.3 ± 33.3	91.5 ± 27.7	.96

Abbreviations: BMI, body mass index; WC, waist circumference; HC, hip circumference; WHR, wait‐to‐hip ratio; NC, neck circumference, IHL, intrahepatic lipids. All data are presented as mean ± SD.

Bold values indicates significance of < .05.

### 3.1 Liver outcomes

Following 12 weeks of dietary intervention, there was a modest and statistically significant improvement in IHL in the LFD group representing approximately a 17% reduction on the log scale, (*p* = .02). In comparison, there was a non‐significant reduction in the MedDiet group (−8%, *p* = .07). There was no significant difference between‐groups even after adjusting for baseline values, diabetes status and visceral fat (*p* = .865). (Table 2). There was also no significant difference for LSM between‐groups (*p* = .58) while within‐group changes were also not significant (MedDiet: 7.8 ± 4.0 to 7.6 ± 5.2, *p* = .43; LFD: 11.8 ± 14.3 to 10.8 ± 10.2, *p* = .99). Liver enzymes including serum ALT, AST and GGT levels were not statistically significant following the MedDiet, however, significant reductions were noted in the LFD group, and the changes observed between‐groups were significant (Table 3).

**TABLE 2:** Estimated differences^a^ between study groups on outcomes at mid‐ and end‐intervention

	Mid‐intervention			End‐intervention		
	*B* ^b^	(95% CI)	*p*‐value	*B*	(95% CI)	*p*‐value
Liver outcomes						
IHL (%)	—	—	—	−.03	(−0.33, 0.28)	.87
LSM (kPa)	—	—	—	−.06	(−0.28, 0.16)	.58
Insulin resistance and biomarkers						
HOMA‐IR	−0.08	(−0.28, 0.12)	.44	0.17	(−0.07, 0.41)	.17
Glucose (mmol/L)	−0.02	(−0.10, 0.06)	.57	−0.04	(−0.13, 0.06)	.43
Insulin (mIU/L)	−0.07	(−0.23, 0.09)	.42	0.20	(−0.01, 0.42)	.067
ALT (U/L)	0.20	(−0.04, 0.43)	.11	**0.31**	**(0.06, 0.56)**	**.017**
AST (U/L)	0.17	(−0.06, 0.40)	.15	0.24	(−0.02, 0.50)	.073
GGT (U/L)	0.09	(−0.12, 0.29)	.40	**0.24**	**(0.01, 0.48)**	**.042**
ALP (U/L)	0.01	(−0.05, 0.08)	.64	0.01	(−0.09, 0.11)	.85
Cholesterol (mmol/L)	0.06	(−0.04, 0.15)	.24	0.06	(−0.02, 0.14)	.17
HDL (mmol/L)	0.09	(−0.02, 0.19)	.11	0.03	(−0.07, 0.13)	.57
LDL (mmol/L)	0.01	(−0.11, 0.13)	.86	0.04	(−0.07, 0.14)	.48
Triglycerides (mmol/L)	−0.03	(−0.22, 0.16)	.75	0.04	(−0.13, 0.20)	.67
Total protein (g/L)	0.82	(−1.89, 3.52)	.55	−0.25	(−2.32, 1.82)	.81
Albumin (g/L)	0.73	(−0.39, 1.85)	.20	**1.05**	**(0.08, 2.02)**	**.035**
Globulin (g/L)	1.00	(−0.93, 2.94)	.31	−0.38	(−2.18, 1.42)	.68
Bilirubin (μmol/L)	0.00	(−0.17, 0.17)	.99	−0.04	(−0.25, 0.16)	.69
hs‐CRP (mg/L)				0.06	(−0.33, 0.45)	.77
Anthropometry and body composition						
Weight	−0.01	(−0.01, 0.00)	.29	0.00	(−0.02, 0.02)	.79
BMI (kg/m^2^)	−0.01	(−0.02, 0.00)	.53	0.00	(−0.02, 0.03)	.74
WC (cm)	0.01	(−0.05, 0.06)	.78	0.03	(−0.02, 0.07)	.30
HC (cm)	−0.02	(−0.08, 0.03)	.45	0.02	(−0.02, 0.05)	.37
Fat mass (%)	−2.22	(−6.13, 1.70)	.27	0.87	(−3.01, 4.75)	.66
Visceral fat (L)	−0.08	(−0.41, 0.26)	.65	0.03	(−0.25, 0.31)	.82
Systolic BP (mmHg)	6.34	(−2.21, 14.88)	.15	4.96	(−2.57, 12.49)	.20
Diastolic BP (mmHg)	5.50	(−0.24, 11.23)	.060	3.76	(−1.75, 9.28)	.18

*Note*: Bold values indicate *p* < .05 where statistical significance was shown.

^a^ Mid‐ and end‐intervention group differences for each outcome were estimated using restricted maximum‐likelihood linear mixed models using outcome data from all available time points. The models were adjusted for pre‐intervention diabetes status and baseline visceral fat level, and implicitly adjusted for baseline levels of the outcome via specification of an unstructured residual error variance–covariance matrix.

^b^ Positive coefficients indicate higher levels of the outcome for the Mediterranean Diet compared to the low‐fat diet. Six outcomes (fat mass, systolic blood pressure, diastolic blood pressure, total protein, albumin, globulin) were analysed in their raw form and thus coefficients reflect estimated mean differences in the outcomes between‐groups; the remaining outcomes were log‐transformed prior to analysis, therefore coefficients for these outcomes reflect estimated mean differences on the log scale.

**TABLE 3:** Descriptive statistics (mean ± standard deviation), within‐groups, for outcome measures in the two study groups at each time point

	Low‐fat diet group (*n* = 23)			Mediterranean diet group (*n* = 19)			Low‐fat diet group *p*‐value^a^	Mediterranean diet group *p*‐value^a^
	Baseline	Mid intervention	End intervention	Baseline	Mid intervention	End intervention		
Liver outcomes								
IHL (%)	9.2 ± 10.7	—	8.9 ± 12.4	13.7 ± 7.8	—	12.1 ± 7.8	**.020**	.069
LSM (kPa)	11.8 ± 14.3	—	10.8 ± 10.2	7.8 ± 4.0	—	7.6 ± 5.2	.99	.43
Insulin resistance and biomarkers								
HOMA‐IR	6.5 ± 5.6	6.6 ± 5.3	5.5 ± 5.5	4.4 ± 3.2	3.4 ± 1.8	3.9 ± 2.3	**<.0005**	.65
Glucose (mmol/L)	6.7 ± 2.0	6.8 ± 1.9	6.8 ± 2.4	5.8 ± 1.6	5.7 ± 1.4	5.7 ± 1.1	.82	.41
Insulin (mIU/L)	20.0 ± 12.4	20.6 ± 13.3	16.4 ± 11.3	16.4 ± 8.9	12.9 ± 4.6	15.5 ± 8.5	**<.0005**	.74
ALT (U/L)	61.5 ± 37.0	45.5 ± 28.5	46.9 ± 20.8	54.1 ± 25.2	54.7 ± 36.5	64.7 ± 39.5	**.009**	.32
AST (U/L)	41.8 ± 21.6	34.7 ± 20.6	34.1 ± 15.4	31.8 ± 12.6	35.0 ± 33.3	39.7 ± 27.4	**.040**	.29
GGT (U/L)	126.7 ± 128.8	90.4 ± 98.8	95.2 ± 72.9	90.1 ± 74.6	75.5 ± 52.3	105.1 ± 91.2	**.029**	.26
ALP (U/L)	93.3 ± 33.3	95.1 ± 30.1	94.2 ± 24.9	91.5 ± 27.7	90.8 ± 25.6	96.8 ± 31.3	.71	.52
Cholesterol (mmol/L)	4.9 ± 1.5	4.8 ± 1.4	4.7 ± 1.4	5.1 ± 1.6	5.1 ± 1.4	5.1 ± 1.6	.084	.72
HDL (mmol/L)	1.2 ± 0.3	1.1 ± 0.2	1.2 ± 0.3	1.2 ± 0.2	1.2 ± 0.3	1.2 ± 0.3	.57	.45
LDL (mmol/L)	2.9 ± 1.3	2.8 ± 1.2	2.8 ± 1.3	3.2 ± 1.3	3.1 ± 1.1	3.1 ± 1.3	.27	.487
Triglycerides (mmol/L)	1.8 ± 0.9	1.8 ± 0.8	1.6 ± 0.7	1.8 ± 0.9	1.8 ± 1.0	1.7 ± 0.8	.41	.49
Total protein (g/L)	77.6 ± 6.1	75.9 ± 5.4	76.5 ± 4.9	75.0 ± 4.7	73.9 ± 5.3	74.2 ± 4.2	.19	.67
Albumin (g/L)	39.1 ± 3.2	38.5 ± 2.3	38.1 ± 2.4	40.4 ± 3.4	39.8 ± 2.6	39.6 ± 1.8	**.027**	.30
Globulin (g/L)	38.5 ± 6.0	37.4 ± 5.7	38.4 ± 5.4	34.6 ± 4.7	34.4 ± 4.6	34.7 ± 4.7	.75	.77
Bilirubin (μmol/L)	12.3 ± 6.7	13.2 ± 6.7	13.1 ± 8.1	16.6 ± 10.5	17.0 ± 9.8	16.7 ± 11.4	.47	.62
hs‐CRP (mg/L)	3.8 ± 2.7	—	3.5 ± 2.6	2.6 ± 2.3	—	2.2 ± 1.9	.31	.89
Anthropometry and body composition								
Weight (kg)	89.8 ± 24.4	91.2 ± 25.9	86.6 ± 19.6	87.7 ± 21.1	87.9 ± 21.5	88.1 ± 21.9	.11	.45
BMI (kg/m^2^)	32.7 ± 6.9	32.6 ± 7.4	31.3 ± 4.9	31.6 ± 5.4	31.4 ± 5.5	31.5 ± 5.5	.16	.63
WC (cm)	108.7 ± 18.9	108.9 ± 20.6	103.9 ± 12.7	105.1 ± 14.7	106.3 ± 16.5	106.2 ± 15.2	.70	.42
HC (cm)	109.8 ± 16.1	112.2 ± 17.3	107 ± 11.4	109.3 ± 14.4	109.5 ± 16.3	110.4 ± 14.2	.64	.95
Fat mass (%)	40.8 ± 7.9	42.8 ± 7.6	38.9 ± 7.9	39.1 ± 7.9	39.0 ± 7.4	38.9 ± 7.7	.35	.96
Visceral fat (L)	4.4 ± 2.1	5.1 ± 2.9	1.5 ± 0.6	3.2 ± 1.5	4.1 ± 2.6	1.8 ± 1.4	**<.0005**	**<.0005**
Systolic BP (mmHg)	127.4 ± 19.2	116.4 ± 14.3	118.6 ± 10.8	125.4 ± 12.1	124.6 ± 13.9	123.3 ± 13.4	**.033**	.52
Diastolic BP (mmHg)	83.3 ± 9.8	79.3 ± 9.9	80.3 ± 8.6	82.8 ± 7.1	84.2 ± 8.3	83.7 ± 8.1	.21	.74

*Note*: Analysed with restricted maximum‐likelihood mixed models. Six outcomes (fat mass, systolic blood pressure, diastolic blood pressure, total protein, albumin, globulin) were analysed in their raw form; the remaining outcomes were log‐transformed prior to analysis. All data are presented as mean ± SD.

Abbreviations: ALP, alkaline phosphatase; ALT, alanine aminotransferase; AST, aspartate aminotransferase; BMI, body mass index; GGT, gamma‐glutamyl transferase; HC, hip circumference; HDL, high‐density lipoprotein; HOMA‐IR, homeostatic model assessment‐insulin resistance; IHL, intrahepatic lipid; LDL, low‐density lipoprotein; LSM, liver stiffness measure; NC, neck circumference; WC, waist circumference; WHR, wait‐to‐hip ratio.

a Within‐group changes from baseline to post‐intervention.

Bold values indicates significance of < .05.

### 3.2 Insulin resistance

There was a significant one‐unit reduction of HOMA‐IR following the LFD (6.5 ± 5.6 to 5.5 ± 5.5, *p* < .01) and a non‐significant 0.5 unit reduction in the MedDiet group (4.4 ± 3.2 to 3.9 ± 2.3, *p* = .07) (Table 2). However, the baseline values for HOMA‐IR were notably lower in the MedDiet group. The differences at the end of the intervention for HOMA‐IR were not significant between‐groups (*p* = .58).

### 3.3 Anthropometry

Within both dietary intervention groups, weight, BMI and waist circumference were not significantly different from pre‐ to post‐intervention. However, the weight reduction in the LFD group almost reached 5% (89.8 ± 24.5 kg to 85.8 ± 18.14 kg, *p* = .382); whereas in those in the MedDiet group gained 1.6 kg (87.7 ± 21.1 to 89.3 ± 22.8, *p* = .63). Visceral fat determined using BIA was reduced significantly in both groups; LFD ([log scale] ‐76%, *p* = <.0005), MedDiet (−61%, *p* = <.0005) although there were no between‐group differences.

### 3.4 Dietary intervention and compliance

The MEDAS score used to assess adherence to the MedDiet and the equivalent score for the LFD were applied to each group’s respective food diaries. Compliance with the MedDiet improved by 2.7 units (6.5 ± 2.0 to 9.2 ± 1.9, out of a maximum possible score of 14) (*p* < .0005). In the LFD group compliance with the prescribed diet improved by 1.0 unit (5.4 ± 2.0 to 6.4 ± 2.3, out of a maximum possible score of 9) (*p* = .035). In the MedDiet at the macronutrient level, there was a non‐significant reduction in energy consumption from 9.2 to 8.4 MJ, this was accompanied by a significant reduction in carbohydrate intake, displaced with an increase in total fat (NS) and a significant increase in MUFAs. Conversely in the LFD, at the macronutrient level, there was a significant reduction in total fat, and while not significant the reduction in energy is of note (8.1 to 7 MJ). However, an in‐depth dietary assessment highlighted further details with respect to compliance with key diet principles. Firstly, there were no significant differences with regard to energy intake following the intervention for either of the dietary arms, as expected given the focus was not on caloric deficit. Wholegrains, fruits and vegetables did not improve within or between‐groups across the intervention period. Baseline intakes for these food groups, as shown in Table 4, were better than expected, compared to the consumption of the general Australian population.^34^ The most prominent changes between‐groups pertained to fat consumption whereby total fat, mono and polyunsaturated fatty acids were significantly increased in the MedDiet group, aligning more closely to dietary intervention targets. This was also affirmed with significant between‐group increases in the MedDiet group at the end of the intervention in long‐chain omega 3's (*p* = .035) and oil equivalents (predominantly because of an increase in extra virgin olive oil) (*p* = .01) in line with MedDiet recommendations.

**TABLE 4:** Dietary intake at baseline and end intervention

	Low‐fat diet		Mediterranean diet		Low‐fat diet group *p*‐value*	Mediterranean diet group *p*‐value*
	Baseline *n* = 23	End intervention *n* = 21	Baseline *n* = 19	End intervention *n* = 18		
Macronutrients						
Energy^a^ (kJ)	8136.5 ± 2752.6	7032.5 ± 2815.8	9204.7 ± 3582.6	8379.2 ± 3176.1	.33	.61
Protein (%E)	19.5 ± 4.0	21.0 ± 5.1	18.2 ± 3.7	20.0 ± 4.2	.12	.28
Carbohydrate (%E)	41.1 ± 7.8	41.0 ± 8.0	43.7 ± 5.7	36.4 ± 7.6	.70	**.001**
Fibre (g)	24.3 ± 11.2	24.0 ± 10.6	24.1 ± 9.7	24.4 ± 9.2	.78	.31
Total fat (%E)	35.3 ± 7.0	32.7 ± 7.8	34.4 ± 5.6	40.1 ± 7.0	**.048**	.09
MUFA (%E)	14.6 ± 3.8	13.4 ± 3.7	14.3 ± 3.6	19.0 ± 4.8	.08	**.047**
PUFA (%E)	5.9 ± 2.5	5.8 ± 1.9	5.8 ± 2.3	7.7 ± 2.4	.20	.21
SFA (%E)	12.1 ± 3.9	10.3 ± 3.4	11.9 ± 2.5	10.8 ± 3.3	.08	.42
Trans fat (g)	1.3 ± 0.8	0.9 ± 0.6	1.2 ± 0.7	0.9 ± 0.5	.23	.26
Alcohol^a^ (g)	1.4 ± 3.1	3.0 ± 5.3	6.3 ± 0.3	5.1 ± 0.3	.44	.38
Micronutrients						
Sodium^a^ (mg)	2410.5 ± 922.4	2202.7 ± 1021.1	2408.61052.1	2077.7 ± 757.9	.98	.14
Potassium^a^ (mg)	3009.6 ± 1195.9	2689.6 ± 957	2865.8 ± 1065.9	2879.6 ± 846.2	.36	.98
Vitamin C^a^ (mg)	137.6 ± 170.8	107.3 ± 91.7	95.0 ± 89.3	96.2 ± 76.7	.46	.95
Calcium (mg)	838.6 ± 359.6	645.6 ± 253.6	861.7 ± 466.6	780.2 ± 354.9	**.025**	.80
Iron (mg)	10.7 ± 4.9	9.7 ± 4.1	11.5 ± 4.6	11.8 ± 4.6	.84	.49
Zinc^a^ (mg)	10.5 ± 4.5	8.9 ± 3.5	10.9 ± 5.0	10.3 ± 2.9	.08	.71
Sugars (g)	83.3 ± 43.7	63.2 ± 26.7	91.6 ± 49.4	67.5 ± 29.6	.40	.09
Caffeine^a^ (mg)	115.3 ± 110.5	102.4 ± 81.6	110.5 ± 91.5	86.1 ± 67.8	.73	.16
Beta carotene equivalents (mg)	2853 ± 3106	2341.4 ± 1572.8	3350.6 ± 3715.4	2656.6 ± 3038.5	.99	.92
F18D2CN6Linoleic (g)	10.9 ± 6.9	9.3 ± 6.4	12.6 ± 8.3	14.4 ± 8.7	.84	.68
F18D3N3 alpha linolenic acid (ALA) (g)	1.5 ± 1.1	1.3 ± 0.8	1.8 ± 1.0	1.7 ± 0.7	.33	.85
F20D5N3 eicosapentaenoic acid (EPA) (g)	0.1 ± 0.1	0.2 ± 0.5	0.1 ± 0.1	0.3 ± 0.3	.94	**.03**
F22D5N3 docosapentaenoic acid (DPA) (g)	0.1 ± 0.1	0.2 ± 0.1	0.1 ± 0.1	0.1 ± 0.1	.96	.14
F22D6N3 docosahexaenoic acid (DHA) (g)	0.1 ± 0.2	0.2 ± 0.5	0.1 ± 0.2	0.5 ± 0.6	.45	.07
Very long chain N3 fatty acids (g)	0.3 ± 0.4	0.5 ± 1.1	0.3 ± 0.4	0.9 ± 0.9	.47	.06
Food groups						
Grains (serve)	6.1 ± 2.9	5.6 ± 3.5	7.9 ± 3.0	6.2 ± 4.1	.20	.06
Wholegrains (serve)	1.1 ± 1.2	2.2 ± 2.1	1.4 ± 1.4	1.8 ± 1.4	.30	.15
Refined grains (serve)	5.0 ± 2.5	3.4 ± 3.1	6.5 ± 3.2	4.4 ± 4.3	.79	**.012**
Vegetables (serve)	4.2 ± 2.9	3.3 ± 2.2	3.3 ± 2.1	3.3 ± 1.7	.94	.91
Dark green vegetables (serve)	0.6 ± 2.0	0.4 ± 0.7	0.5 ± 0.8	0.5 ± 0.6	.88	.95
Tomatoes (serve)	0.5 ± 0.5	0.3 ± 0.4	0.2 ± 0.3	0.6 ± 0.5	**.021**	**.010**
Fruit (serve)	1.6 ± 1.4	1.3 ± 1.4	1.0 ± 0.9	1.0 ± 0.9	.25	.68
Red meats (serve)	0.8 ± 0.8	0.6 ± 0.6	0.8 ± 1.2	0.4 ± 0.6	.22	.67
Poultry (serve)	0.5 ± 0.6	0.7 ± 0.7	0.5 ± 0.4	0.7 ± 1.0	.36	.07
High long chain N3 fatty acid seafoods (serve)	0.1 ± 0.2	0.2 ± 0.6	0.1 ± 0.3	0.4 ± 0.5	.35	**.036**
Legumes (serve)	0.2 ± 0.8	0.3 ± 0.6	0.3 ± 0.6	0.2 ± 0.4	.16	.57
Nuts (serve)	0.5 ± 1.0	0.4 ± 0.5	0.9 ± 1.4	1.0 ± 1.3	.19	.07
Dairy (serve)	1.6 ± 1.0	1.2 ± 0.7	1.7 ± 1.3	1.3 ± 0.8	.33	.68
Oil equivalents (tsp)	8.2 ± 4.8	7.5 ± 4.9	9.5 ± 6.7	13.4 ± 8.3	.66	**.030**
Added sugars (%)	7.6 ± 7.3	4.1 ± 4.4	10.4 ± 10.1	5.4 ± 5.1	**.047**	**.009**
MEDAS dietary compliance scores						
Low‐fat diet score	5.4 ± 2.0	6.4 ± 2.3				**.035**
Mediterranean diet score			6.5 ± 2.0	9.2 ± 1.9		**<.0005**

*Note*: MEDAS Scores* Analysed with restricted maximum‐likelihood mixed models.

Abbreviations: MUFA, monounsaturated fatty acids; N3, omega‐3; PUFA, polyunsaturated fatty acids; SFA, saturated fatty acids.

^a^ Non‐parametric variable.

* Indicates significance within study arm, pre and post‐intervention (*p* < .05).

Bold values indicates significance of < .05.

### 3.5 Sensitivity analysis

Fourteen of the between‐group models were refitted with a single potentially influential observation (not always the same participant) removed from each. In these models, the results were largely unchanged in terms of overall interpretation, however, in contrast to the primary analysis there was statistically significantly higher HOMA‐IR values (*B* = 0.28 [95%% CI: 0.05, 0.51], *p* = .015) for MedDiet compared to LFD at end‐intervention.

## 4 DISCUSSION

The aim of this RCT was to determine whether the MedDiet was more beneficial compared to an LFD in patients with NAFLD in relation to several metabolic parameters including intrahepatic lipids and insulin resistance, as well as LSM and other parameters of health. Furthermore, the study aimed to assess the relationship between changes in the above metabolic factors and body composition across the two dietary patterns. Importantly, the results of this RCT showed that MedDiet did not significantly reduce hepatic steatosis and was not superior to LFD in an Australian free‐living NAFLD cohort. Indeed, we found the LFD significantly reduced hepatic steatosis and HOMA‐IR in NAFLD subjects following the 12‐week intervention, in contrast to MedDiet that led to non‐significant albeit clinically relevant, reductions in both metabolic parameters. These changes however were not significant when compared between‐groups. However, an important finding from the study was that both dietary interventions resulted in significant reductions in visceral fat, with the reduction being more pronounced in the LFD group.

These findings align with evidence that weight loss elicits improvements in hepatic steatosis with the LFD group achieving almost 4% weight loss, in 12 weeks despite the intervention having an emphasis on weight maintenance. In contrast, although there were potentially, clinically meaningful improvements in hepatic steatosis in the MedDiet group despite remaining essentially weight stable, these were not statistically significant. This reiterates that energy restriction and subsequently weight loss appear to play a central role in the management of NAFLD. However, weight loss is difficult to achieve and hard to maintain and what this study indicates is that changes in dietary behaviours such as choosing foods that improve dietary quality may be sustainable and lead to small but meaningful effects in NAFLD. Still, current EASL–EASD–EASO Clinical Practice Guidelines for the management of non‐alcoholic fatty liver disease recommend the MedDiet as the optimal dietary pattern.^35^ In comparison, the American Association for the Study of Liver Diseases (AASLD) guidelines are more conservative recommending that additional rigorous, prospective studies such as ours and longer‐term data with histological endpoints are required before specific macronutrient compositions can be recommended.^36^ Although the AASLD wording only acknowledges macronutrients, dietary patterns which represent the whole diet, are needed to make sustainable lifestyle changes. Other guidelines for NAFLD agree with those of AASLD and EASL, APASL clearly highlights the importance of a hypocaloric diet. It is important to note that such guidelines are based on studies which are predominantly conducted in Mediterranean countries, and it seems the application of MedDiet in a multiethnic, diverse Western population such as in the present study is less straightforward. For example, to better understand the context and implications of adding energy‐dense foods to a multiethnic diet and how this can be balanced ad libitum or to achieve a caloric deficit. Furthermore, issues such as length of dietary intervention and additional participant support(s) to achieve higher levels of compliance are needed in multiethnic non‐Mediterranean populations to obtain the full translational benefits of a MedDiet.

There have been few studies showing significant beneficial effects with the provision of MedDiet in Western populations with NAFLD. Ryan et al conducted a cross over RCT under tightly controlled conditions including the full provision of diet.^14^ This important proof of concept study showed that the MedDiet does indeed improve hepatic and metabolic outcomes in participants with NAFLD. However, under less stringent conditions, as shown in the present RCT in free‐living adults, it appears that more attention is warranted on how to apply and therefore translate the MedDiet into a population who does not habitually follow this dietary pattern. Furthermore, the addition of extra virgin olive oil high in mono‐unsaturated fatty acids, antioxidants, fat‐soluble vitamins and polyphenols is believed to be one of the key elements providing health benefits in the MedDiet, and yet is energy dense, and therefore adding it to a predominantly Western Diet can lead to higher overall energy intake, as seen in this study, albeit weight remained stable. Furthermore, the results from this study are reinforced by another study conducted in Western Australia where ad libitum MedDiet and LFD both improved hepatic steatosis indicating improvement in overall diet quality regardless of dietary prescription was beneficial.^15^ The present study where the LFD group achieved almost 4% weight reduction contrasts with the former study where no changes in weight were observed. While the reduction in total energy consumption did not reduce significantly within the LFD the change is noteworthy and with adequate power for secondary outcomes such a reduction may have reached significance. Furthermore, a modest reduction in total fat intake in the LFD group shows a further improvement in diet quality at the macronutrient level.

In the LFD group, there were meaningful reductions in weight, waist circumference and BMI. However, this study was not sufficiently powered for these secondary outcomes, and thus these changes were not statistically significant. Nonetheless robust evidence from a meta‐analysis of eight randomised controlled trials reported that 5% weight loss has been shown to improve hepatic outcomes.^37^ This meta‐analysis is in agreement with the results from this study for the low‐fat diet group where weight loss resulted in a significant reduction in hepatic steatosis. The reduction in visceral fat observed in the LFD group was also not surprising given the weight loss achieved. Of interest, however, the MedDiet group also saw a substantial and significant reduction in visceral fat after 12 weeks despite this group remaining largely weight stable. This supports the evidence that MedDiet can elicit favourable changes in body composition in the absence of weight loss.^38^ To explain this, another study comparing three ad libitum diets in overweight individuals for 6 months concluded that diet composition had no significant effect on preventing weight regain. The authors did, however, find that a diet rich in fat, especially MUFAs resulted in less body fat accumulation compared to the control diet.^39^ This is in line with other studies showing components of a MedDiet including MUFA are inversely associated with abdominal adipose tissue accumulation regardless of body weight.^40^ The mechanism for such benefits is largely unknown, however may be, at least in part, explained by the overall improvement in diet quality associated with increased MUFA intake, as many high MUFA foods and nutrient and antioxidant rich. In this small sample, the reduction in visceral fat after 12 weeks of MedDiet did not lead to statistically significant reductions in hepatic steatosis and HOMA‐IR although our observation of a clinically relevant 0.5 unit reduction in HOMA‐IR is not dissimilar to other studies in which a reduction of 0.8 units in HOMA‐IR with metformin and diet was demonstrated over 9 months.^41^ While our study was powered to see a decrease in hepatic steatosis with the dietary patterns, the effect size in metabolic parameters between‐groups was not large enough to see a significant between‐group difference.

Mediterranean diet studies in NAFLD are limited in non‐Mediterranean countries such as Australia and our results showing a lack of superiority of MedDiet over LFD need to be carefully considered alongside other studies. For example, the lack of difference could be because the LFD was more familiar as participants may have potentially received advice on this diet before and in addition may have perceived these foods to be more freely available. The literature showing Mediterranean diet is beneficial, while demonstrated several times has not been shown to be superior in free living, Western or indeed Australian cohorts.^15^ The changes in dietary adherence, as indicated by the MEDAS score were also marginal, and this too could explain the modest changes seen. However, it does appear that the MedDiet contributed to improving diet quality and in metabolic outcomes such as visceral fat reduction even in the absence of weight loss. Given the paucity of studies assessing the effects of a MedDiet alongside weight loss targets are lacking in NAFLD subjects, prospective studies are needed to determine if there are combined benefits with regard to improved diet quality and weight loss, beyond the LFD. Such studies are needed a priori to inform dietary recommendations and guidelines for NAFLD in the absence of effective pharmaco‐therapy.

The main strengths of this study are its randomised controlled trial design which was carried out in free‐living adults with NAFLD. Furthermore, the participants all had a matched number of face‐to‐face and phone appointments, controlling for any biases around contact with a healthcare professional. We also included robust collection of a 3‐day food diary to measure dietary compliance which was validated by an accredited practising dietitian who also provided telehealth support between face‐to‐face appointments. The MEDAS score was also used to measure compliance with MedDiet and LFD. Hepatic outcomes were also robust including the gold standard MRS approach for IHL quantification, as well as Fibroscan for liver stiffness measure. Still, the limitations of this study include its small sample size which despite being powered on hepatic steatosis, with the unintended weight loss in the LFD group the effect size was not sufficient to assess between‐group differences. The small sample size also limits generalizability of the secondary outcomes presented herein. Using BIA for assessing visceral fat has shown conflicting evidence with regard to validity and is less accurate in obese individuals and thus higher quality imaging such as CT or MRI should be used to verify these findings in future studies.^42^, ^43^, ^44^ Furthermore, in dietary interventions it is not possible to blind clinicians and participants from dietary prescription. Despite being comparable in duration to similar studies^15^, ^17^, ^45^ longer duration (or term) studies are needed to show efficacy, particularly in relation to outcomes such as LSM and to determine the sustainability of dietary interventions and impact on dietary behaviours. The shorter duration may also explain why some parameters which showed clinically meaningful improvement were not statistically significant. Baseline diets in this study cohort were also not in line with the “poor” quality intake often seen in individuals with NAFLD,^46^ and future studies may consider screening participants and recruiting those with poor diet quality at baseline. Finally, the baseline differences between study groups, despite randomisation may have had some impact on scope for changes with high glucose, visceral fat and heart rate in the LFD group. Diets overall were better than that of the general Australian population, also potentially hindering the scope to improve diet quality and thus outcomes in only 12 weeks.

## 5 CONCLUSION

In this RCT in NAFLD subjects, there were no significant differences in IHL and metabolic parameters observed between‐groups for the MedDiet compared to LFD. However, in the LFD arm there were significant reductions in IHL and metabolic parameters whereas these improvements were not significant in the MedDiet group. Significant improvements in visceral fat were seen in both groups. This study highlights that the provision of dietary interventions in free‐living adults with NAFLD is challenging (ACTRN12615001010583).

## CONFLICT OF INTEREST

All authors have no perceived conflict of interest to declare.

## AUTHOR CONTRIBUTION

ESG and ACT conceptualised the study. ESG designed the intervention and drafted the manuscript. All authors provided intellectual input into the manuscript and approved the final version.
